# The S-Layer Homology Domain-Containing Protein SlhA from *Paenibacillus alvei* CCM 2051^T^ Is Important for Swarming and Biofilm Formation

**DOI:** 10.1371/journal.pone.0076566

**Published:** 2013-09-18

**Authors:** Bettina Janesch, Andrea Koerdt, Paul Messner, Christina Schäffer

**Affiliations:** NanoGlycobiology Unit, Department of NanoBiotechnology, Universität für Bodenkultur Wien, Vienna, Austria; University of Illinois at Chicago College of Medicine, United States of America

## Abstract

**Background:**

Swarming and biofilm formation have been studied for a variety of bacteria. While this is well investigated for Gram-negative bacteria, less is known about Gram-positive bacteria, including *Paenibacillus alvei*, a secondary invader of diseased honeybee colonies infected with *Melissococcus* *pluton* , the causative agent of European foulbrood (EFB).

**Methodology:**

*Paenibacillus alvei* CCM 2051^T^ is a Gram-positive bacterium which was recently shown to employ S-layer homology (SLH) domains as cell wall targeting modules to display proteins on its cell surface. This study deals with the newly identified 1335-amino acid protein SlhA from *P. alvei* which carries at the C‑terminus three consecutive SLH-motifs containing the predicted binding sequences SRGE, VRQD, and LRGD instead of the common TRAE motif. Based on the proof of cell surface location of SlhA by fluorescence microscopy using a SlhA-GFP chimera, the binding mechanism was investigated in an *in vitro* assay. To unravel a putative function of the SlhA protein, a knockout mutant was constructed. Experimental data indicated that one SLH domain is sufficient for anchoring of SlhA to the cell surface, and the SLH domains of SlhA recognize both the peptidoglycan and the secondary cell wall polymer *in vitro*. This is in agreement with previous data from the S-layer protein SpaA, pinpointing a wider utilization of that mechanism for cell surface display of proteins in *P. alvei*. Compared to the wild-type bacterium Δ*slhA* revealed changed colony morphology, loss of swarming motility and impaired biofilm formation. The phenotype was similar to that of the flagella knockout Δ*hag*, possibly due to reduced EPS production influencing the functionality of the flagella of *ΔslhA*.

**Conclusion:**

This study demonstrates the involvement of the SLH domain-containing protein SlhA in swarming and biofilm formation of *P. alvei* CCM 2051^T^.

## Introduction

The constitution of the cell surface of bacteria strongly influences the physicochemical properties of bacterial cells, the bacterial life-style (planktonic versus biofilm) and the potential for survival in a competitive habitat. Consequently, investigating cell surface compounds of bacteria, their display mechanism and their functional influence on the bacterium can add to our knowledge of strategies for interfering with bacterial colonization as relevant, for instance, in the context of combating bacterial infections.

S-layer homology (SLH) domains are cell wall-targeting modules employed by various Gram-positive as well as Gram-negative bacteria to display extracellular proteins such as enzymes, outer membrane proteins, and surface (S-) layer (glyco) proteins on the bacterial cell surface [1,2]. S‑layers are 2D crystalline arrays that completely cover bacterial cells [3] that are in many bacteria of the *Bacillaceae* family, non-covalently attached to the bacterial cell envelope via their SLH-domains [4,5]. While lectin type-like binding of SLH-domains to a peptidoglycan (PG)-associated, non-classical, pyruvylated secondary cell wall polymer (SCWP) has been known for some time [2,6], we have shown recently for the S‑layer protein SpaA of the Gram-positive bacterium *Paenibacillus alvei* CCM 2051^T^ that its SLH-domains have dual recognition function [2]: The SLH-domains recognize a SCWP with the structure [(Pyr4,6)-β-D-ManpNAc-(1→4)-β-D-GlcpNAc-(1→3)]_n~11_ -(Pyr4,6)-β-D-ManpNAc-(1→4)-α-D-GlcpNAc-(1→ that is linked via a phosphate-containing bridge to muramic acid residues of the PG backbone [7], and PG itself [2]. Furthermore, two out of three functional SLH-domains were found to be sufficient for cell wall binding of SpaA, regardless of the location of the SLH-domains [2].

**Figure 1:**
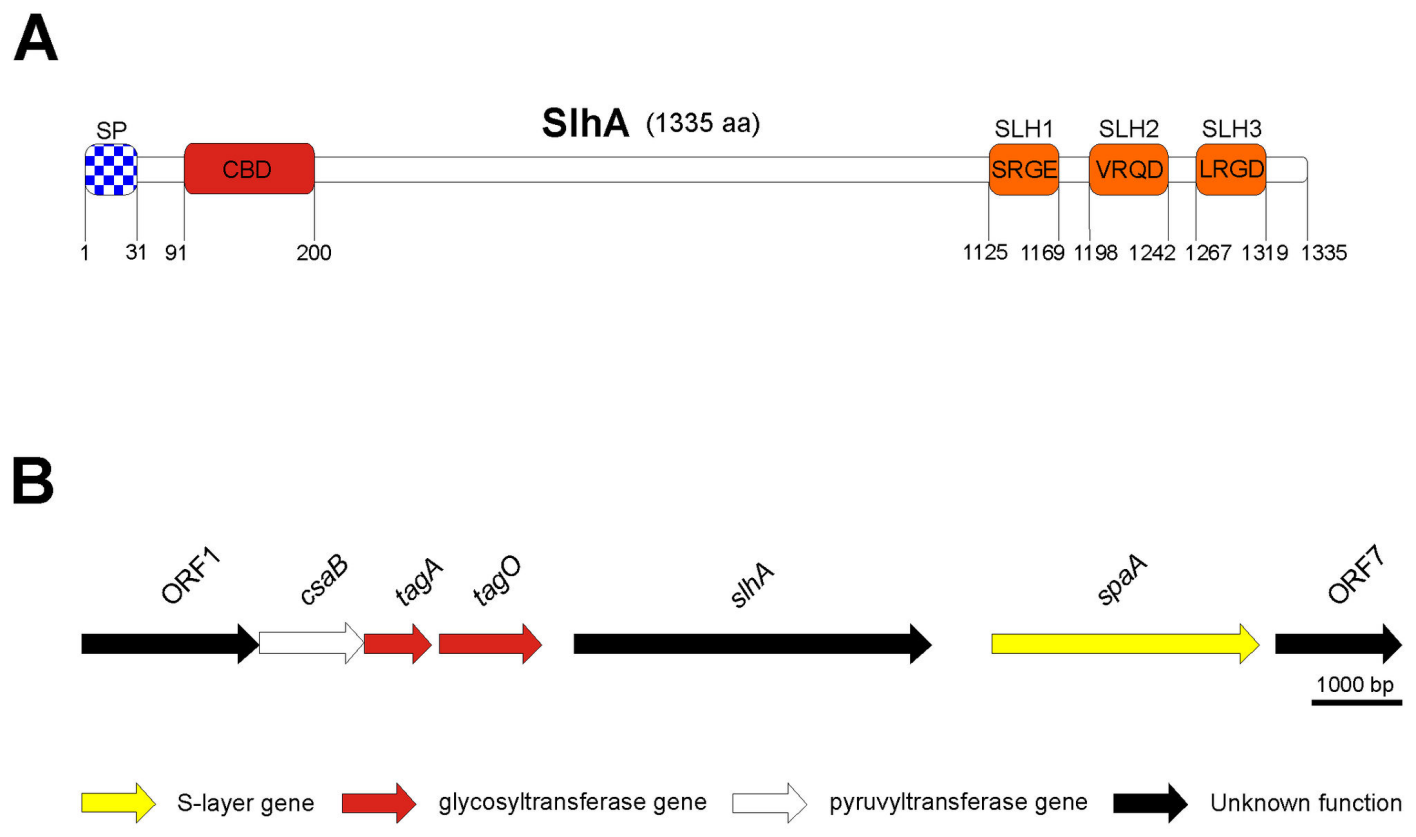
Schematic drawing of the SlhA protein (A) and genetic localization of the *slhA* gene in the SCWP biosynthesis locus of *P. alvei* CCM 2051^T^ (B). bp, base pairs; aa, amino acids; SP, signal peptide; CBD, carbohydrate binding domain; SLH, S-layer homology domain.

In addition to the S‑layer protein, the *P. alvei* CCM 2051^T^ genome reveals a suite of at least 17 more open reading frames encoding SLH-domains. Among these presumably surface located proteins is a protein named SlhA (S‑layer homology domain protein A) that is the focus of the current study. SlhA is a 1335-amino acid protein showing only 17% of overall homology to SpaA. It comprises a typical Gram-positive N-terminal signal peptide (residues 1-31 of the pre-protein) followed by a galactose-binding domain (CBM6, residues 91-200) typical of proteins binding to specific ligands, such as cell-surface-attached carbohydrates, and three C-terminal SLH-domains containing the predicted modified binding motifs SRGE in SLH-domain 1 (residues 1125-1169), VRQD in SLH-domain 2 (residues 1198-1242), and LRGD in SLH-domain 3 (residues 1267-1319) (Figure 1A). Thus, the question arose, if the cell surface display mechanism established for the SpaA S‑layer protein [2] would also be valid for SlhA. It is important to note that while the highly conserved four amino acid motif TRAE present in many SLH-domains [8] plays a key role for the binding function to SCWP [8,9], functional variations in that motif have been reported for *P. alvei* CCM 2051^T^ where the motifs TVEE and TRAQ are present [2] as well as for *Thermoanaerobacterium* *thermosulfurigenes* EM1 [8].

The *slhA* gene of *P. alvei* CCM 2051^T^ is located on the bacterial chromosome between the predicted SCWP biosynthesis locus (downstream) and the S‑layer gene *spaA* (upstream) where it is transcribed under its own promoter (Figure 1B) [10]. Since this, together with its prominent location on the bacterial cell surface, might be indicative of the involvement of the SlhA protein in proper cell envelope formation and/or mediation of cell surface phenomena in *P. alvei* CCM 2051^T^ as potentially important to the life-style of the bacterium, we were interested in further characterizing this protein.

Regarding its life-style, *P. alvei* CCM 2051^T^ is a mesophilic, endospore-forming bacterium that has been reported to swarm vigorously on solidified standard culture medium [11,12]. Swarming is a flagella-driven strategy for motility utilized by a wide range of bacterial species, including paenibacilli [13,14]. While swarming has been previously regarded as a preliminary step in biofilm formation [15], it is now evident that it can be antagonistically regulated [16-18]. For SadB (surface attachment defective B) from *Pseudomonas aeruginosa*, for instance, it has been shown that swarming and biofilm formation are inversely regulated via the modulation of flagellar reversals and by influencing the production of exopolysaccharides [16-18]. In a bacterial biofilm, cells are tightly associated with each other and/or attached to a surface and embedded in a self-produced matrix of exopolymeric substances (EPS) [15,19-21]. This matrix contains a variety of (glyco) proteins, (glyco) lipids, exopolysaccharides and, in several cases, extracellular DNA [22]. It is crucial for preventing the washout of enzymes, keeping them close to cells and allowing for effective degradation of polymeric and particulate material. Since the biofilm confers several advantages to the bacteria, such as increased resistance against toxic conditions or increased ability to escape the immune response of the host [23,24], it is the preferred life-style of many, especially pathogenic, bacteria. Among *Paenibacillus* species, *Paenibacillus polymyxa*, a growth-promoting rhizobacterium, is one of the best studied organisms regarding biofilm formation [25]. While the possibility to knock-out *slhA* demonstrated that the SlhA protein is not necessary for viability of *P. alvei* CCM 2051^T^ cells (this study), we speculated that it might affect bacterial motility.

In this study, we have investigated the predicted cell surface protein SlhA from *P. alvei* CCM 2051^T^. This included (i) demonstration of its co-display with the S‑layer protein SpaA on the bacterial cell surface *in vivo* by homologous co-expression of a chimera made of SlhA-enhanced green fluorescent protein (EGFP) and His_6_-tagged SpaA, (ii) assessment of the relevance of the SLH-domains of SlhA for cell surface anchoring in an *in vitro* binding assay using His_6_-tagged SlhA variants and native PG-containing cell wall sacculi that either contained SCWP or were deprived of it, and (iii) first functional insights into SlhA. In accordance with previous data from the S-layer protein SpaA our experiments indicated a dual recognition function of SlhA for both PG and SCWP with the innermost SLH-domain being sufficient for cell surface anchoring. This pinpoints a wider utilization of this mechanism for cell surface display of proteins in *P. alvei* CCM 2051^T^. The *slhA* knockout mutant displayed changed colony morphology, lost its ability to swarm and showed impaired biofilm formation, possibly due to changes in the EPS. In contrast, a mutation that disrupted flagella synthesis did not show altered EPS production or colony morphology.

## Materials and Methods

### Bacterial strains and growth conditions

*P. alvei* CCM 2051^T^ was obtained from the Czech Collection of Microorganisms (CCM; Brno, Czech Republic) and was grown at 37°C and 200 rpm in Luria-Bertani (LB) broth or on LB agar plates supplemented with 10 µg/ml chloramphenicol (Cm), when appropriate. *Escherichia coli* DH5α cells (Invitrogen) were cultivated in selective Luria Bertani (LB) medium (agar and broth) supplemented with 30 μg/ml chloramphenicol (Cm). All strains used in this study are listed in Table 1.

**Table 1:** Bacterial strains and plasmids used in this study.

**Strain or plasmid**	**Genotype and/or relevant characteristics**	**Source**
*Paenibacillus alvei* CCM 2051^T^	wild-type isolate; Km^r^	Czech Collection of Microorganisms (CCM)
*Escherichia coli* DH5α	F^-^ϕ80d*lacZ* M15 (*lacZYA-argF*)*U169 deoR recA1 endA1 hsdR17* (rK^-^mK^-^) *phoA supE44 thi-1 gyrA96 relA1* ^-^	Invitrogen
*Escherichia coli* BL21 (DE)	*F* ^*-*^ *, ompT, hsdS* (*rB* ^*-*^ *mB* ^*-*^), *gal, dcm* (DE3)	Invitrogen
*P. alvei* CCM 2051^T^ ∆*slhA*	*P. alvei* CCM 2051^T^ carrying a targetron insertion at the *slhA* locus; Cm^r^	This study
*P. alvei* CCM 2051^T^ ∆*hag*	*P. alvei* CCM 2051^T^ carrying a targetron insertion at the *hag* locus; Cm^r^	This study
*P. alvei* CCM 2051^T^ ∆*slhA*_comp_	*P. alvei* CCM 2051^T^ carrying a targetron insertion at the *slhA* locus and the pEXALV_P(SlhA)_SlhA plasmid; Cm^r^	This study
*P. alvei* CCM 2051^T^ (pEXALV)	*P. alvei* CCM 2051^T^ wild-type isolate carrying the pEXALV plasmid; Cm^r^	This study
*P. alvei* CCM 2051^T^ ∆*slhA* (pEXALV)	*P. alvei* CCM 2051^T^ carrying a targetron insertion at the *slhA* locus and the pEXALV plasmid; Cm^r^	This study
*P. alvei* CCM 2051^T^ ∆*slhA* (pSURF)	*P. alvei* CCM 2051^T^ carrying a targetron insertion at the *slhA* locus and the pSURF co-display plasmid; Cm^r^	This study
pEXALV	pNW33N carrying the *sgsE* S-layer gene promoter of *G* *. stearothermophilus* NRS 2004/3a; Cm^r^	[26]
pET28a	Expression vector with a His_6_-tag, Kan^r^	Novagen
pTT_*wsfA*243	pTT_*wsfP*1176 targeted for insertion at position 243/244 from the initial ATG of *wsfA*	[28]
pTT_*SlhA*351	pTT_*wsfA*243 targeted for insertion at position 351/352 from the initial ATG of *slhA*	This study
pET28a_SlhA_6His	pET28a carrying the His_6_-tagged *slhA* gene of *P. alvei* CCM 2051^T^; Kan^r^	This study
pET28a_SlhA_SLH12_6His	pET28a carrying a His_6_-tagged *slhA* gene were SLH domain 3 was eliminated by truncation; Kan^r^	This study
pET28a_SlhA_SLH1_6His	pET28a carrying a His_6_-tagged *slhA* gene were SLH domain 2 and 3 were eliminated by truncation; Kan^r^	This study
pET28a_SlhAdSLH_6His	pET28a carrying a truncated His_6_-tagged *slhA* gene *slhA* gene of *P. alvei* CCM 2051^T^; Kan^r^	This study
pEXALV_P(SlhA)_SlhA	pEXALV carrying the *slhA* gene and the promoter of *slhA*; Cm^r^	This study
pEXALV_EGFP	*P. alvei* CCM 2051^T^ carrying *egfp*; Cm^r^	[2]
pEXALV_SP_SpaA_6HIS	pEXALV carrying the his-tagged *spaA* gene of *P. alvei* CCM 2051^T^	[10]
pEXALV_SlhA_Linker_EGFP	pEXALV carrying a fusion construct of *slhA* and *egfp*; Cm^r^	This study
pSURF	pEXALV carrying a fusion construct of *slhA* and *egfp* and the his-tagged *spaA* gene of *P. alvei* CCM 2051^T^; Cm^r^	This study

### General molecular methods

All enzymes were purchased from Fermentas. Genomic DNA of *P. alvei* CCM 2051^T^ was isolated by using a Genomic Tip 100 kit (Qiagen) according to the manufacturer’s instructions, except that cells were broken by repeated freezing and thawing cycles (10 times) [26]. The GeneJET™ Gel Extraction Kit (Fermentas) was used to purify DNA fragments from agarose gels and to purify digested plasmids and oligonucleotides. Plasmid DNA from transformed cells was isolated with the GeneJET™ Plasmid Miniprep kit (Fermentas). Agarose gel electrophoresis was performed as described elsewhere [27]. Primers for PCR and DNA sequencing were purchased from Invitrogen (Table 2). PCR was performed using the Phusion^®^ High-Fidelity DNA Polymerase (Fermentas) and the thermal cycler My Cycler^TM^ (Bio-Rad). Transformation of chemically competent *E. coli* DH5α cells was done according to the manufacturer’s protocol (Invitrogen). Transformation of *P. alvei* CCM 2051^T^ wild-type or *P. alvei* CCM 2051^T^ Δ*slhA* cells, with the latter showing a better transformation efficiency, is described elsewhere [26]. Transformants were screened by PCR using RedTaq ReadyMix PCR mix (Sigma-Aldrich), and recombinant clones were analyzed by restriction mapping and confirmed by sequencing (LGC).

**Table 2:** Oligonucleotide primers used for PCR amplification reactions.

**Primers**	**Sequence (5’ → 3’)^a^**
SlhA_351|352a-IBS	AAAAAAGCTTATAATTATCCTTAAGGGTCCAACTGGTGCGCCCAGATAGGGTG
SlhA_351|352a-EBS1d	CAGATTGTACAAATGTGGTGATAACAGATAAGTCCAACTGTATAACTTACCTTTCTTTGT
SlhA_351|352a-EBS2	TGAACGCAAGTTTCTAATTTCGATTACCCTTCGATAGAGGAAAGTGTCT
SlhA_KO_for1	ACGGCTAACATTATGATCAATGGTTCG
SlhA_KO-rev2	GCGACACCAACACTAGTCGGCTTC
Flag_738|739s-IBS	AAAAAAGCTTATAATTATCCTTAAAAGACATGTCCGTGCGCCCAGATAGGGTG
Flag_738|739s-EBS1d	CAGATTGTACAAATGTGGTGATAACAGATAAGTCATGTCCGATAACTTACCTTTCTTTGT
Flag_738|739s-EBS2	TGAACGCAAGTTTCTAATTTCGGTTTCTTTCCGATAGAGGAAAGTGTCT
Flag_SphI_for	aatcaGCATGCGTATTAATCACAATATCAGCTC
Flag_KpnI_6His_rev	aatcaGGTACCTTAATGGTGATGGTGATGGTGACGGAGCAATTGCAGTACTCCTTGAGG
SlhA_ohne SP_NcoI_for	aatcCCATGGCAAGCGCCAACCATTTCGTATTTC
SlhA_ganz_XhoI_rev	aatcCTCGAGTTAATGGTGATGGTGATGGTGCATCTTAGGCAG
SlhA_His_SLH12_XhoI_rev	aatcCTCGAGTTAATGGTGATGGTGATGGTGCGCAAGCATGACTGCTGCATCC
SlhA_His_SLH1_XhoI_rev	aatcCTCGAGTTAATGGTGATGGTGATGGTGCGGAATTTCCTTCGCACGAACG
SlhA_His_ohne SLH_XhoI_rev	aatcCTCGAGTTAATGGTGATGGTGATGGTGCATCTTACCAACTACATAGTATCC
SlhA_SphI_for	aatcGCATGCAGAAACTGGTATCC
SlhA_ohne Stop_Linker_XbaI_rev	aatcTCTAGATCCAGCACCGCCGGCACCCATCTTAGGCAGTTTCTTCAAATC
P(SgsE)_SacI_for	aatcaGAGCTCTGTTTTTGCACAAAATGTTTGCCAACC
SpaA_6His_SacI_rev	aatcaGAGCTCTTAATGGTGATGGTGATGGTGCTTACCGGAGTATGTTCCAGG
P(SlhA)_HindIII_for	aatcaAAGCTTCGATTACCACAATTATTACATGCGG
SlhA_KpnI_rev	aatcGGTACCTTACATCTTAGGCAGTTTC

a Artificial restriction sites are underlined. Lowercase letters indicate artificially introduced bases to improve restriction enzyme cutting.

### Gene knockouts

Disruption of the *slhA* gene located in the SCWP biosynthesis locus of *P. alvei* CCM 2051^T^ and the flagella gene (*hag*, PAV_2c01710) was performed as described previously [26]. The Ll.LtrB targetron of pTT_wsfA243 was retargeted prior to transformation into *P. alvei* CCM 2051^T^. Identification of potential insertion sites and design of PCR primers for the modification of the intron RNA was accomplished by a computer algorithm (www.Sigma-Aldrich.com/Targetronaccess). The retargeted Ll.LtrB targetron was subsequently digested with HindIII and BsrGI and ligated into pTT_wsfA243 [28] digested with the same restriction enzymes, thereby replacing the *wsfA* targetron.

To complement the knockout, *slhA* under its native promoter was amplified from *P. alvei* CCM 2051^T^ genomic DNA using the primers P(SlhA) _HindIII_for/ SlhA_KpnI_rev. The amplification product was SphI/KpnI-digested and inserted into the linearized vector pEXALV. The resulting plasmid was named pEXALV_P(SlhA) _SlhA (Table 1) and was transformed into *P. alvei* CCM 2051^T^ Δ*slhA* cells [26].

### Plasmid construction for *in vivo* studies

For analyzing co-display of SlhA and SpaA on *P. alvei* CCM 2051^T^ Δ*slh*A cells *in vivo*, the vector pSURF was designed based on the pEXALV_EGFP vector [2]. For the construction of a chimera of SlhA and enhanced green fluorescent protein (EGFP) including a GAGGAGGT linker, the *slhA* gene was PCR-amplified from genomic DNA of *P. alvei* CCM 2051^T^ using the primer pair SlhA_SphI_for/ SlhA_w/o Stop_Linker_XbaI_rev. The amplification product was SphI/XbaI-digested and inserted into linearized pEXALV_EGFP. The resulting plasmid was named pEXALV_SlhA_Linker_EGFP (Table 1). Subsequently, the *sgsE* S‑layer gene promoter of *G* *. stearothermophilus* NRS 2004/3a, P(SgsE), and the S‑layer gene *spaA* of *P. alvei* CCM 2051^T^ were amplified from pEXALV_SP_SpaA_6HIS as a template using the primers P(SgsE) _SacI_for/ SpaA_6His_SacI_rev and ligated into pEXALV_SlhA_Linker_EGFP. The resulting plasmid was named pSURF (Table 1).

### Recombinant production of His-tagged SlhA variants for *in vitro* assays

All SlhA variants used for *in vitro* assays were produced as His_6_-tagged constructs for detection purposes. The C-terminal His_6_-tag was fused to the sequence of *slhA* by PCR, using the primers SlhA_ohne SP_NcoI_for/ SlhA_ganz_XhoI_rev for *slhA* lacking the sequence coding for the signal peptide, SlhA_ohne SP_NcoI_for/ SlhA_His_SLH12_XhoI_rev for *slhA* lacking the sequences coding for the signal peptide and the third SLH domain, SlhA_ohne SP_NcoI_for/ SlhA_His_SLH1_XhoI_rev for *slhA* lacking the sequences coding for the signal peptide, the second and third SLH domain, and SlhA_ohne SP_NcoI_for/ SlhA_His_ohne SLH_XhoI_rev for *slhA* lacking the sequences coding for the signal peptide and all three SLH domains.

All amplifications were done using *P. alvei* CCM 2051^T^ genomic DNA as a template. The different His_6_-tagged *slhA* amplification products were digested with NcoI/XhoI and cloned into NcoI/XhoI-linearized pET28a(+) (Novagen). The corresponding plasmids were named pET28a_SlhA_6His (encoding native SlhA lacking the SP; designation: SlhA recombinant), pET28a_SlhA_SLH12_6His (encoding SlhA lacking the SP and SLH domain 3; designation: SlhA_12_), pET28a_SlhA_SLH1_6His (encoding SpaA lacking the SP and SLH domains 2 and 3; designation: SlhA_1_), pET28a_SlhAdSLH_6His (encoding SpaA lacking the SP and SLH domains 1, 2, and 3; designation: SlhA w/o SLH) (Table 1; compare with Figure 1A) and transformed into *E. coli* BL21 (DE3) cells. Freshly transformed cells were grown in LB medium [27] to the mid exponential growth phase (OD_600_ ~0.6), protein expression was induced with a final concentration of 0.5 mM isopropyl-β-D-thiogalactopyranosid (IPTG) and cultures were grown for additional 4 h at 37°C and 200 rpm. Cells were harvested by centrifugation (4,500 x *g*, 30 min, 4°C).

### Preparation of native peptidoglycan-containing cell wall sacculi

For the preparation of protein-free peptidoglycan (PG)-containing cell wall sacculi including native SCWP of *P. alvei* CCM 2051^T^ (PG(+)), cells were grown until the late exponential phase and broken by ten freezing and thawing cycles [26]. After centrifugation (35,000 x *g*, 30 min, 4°C), the pellet was resuspended in 50 mM Tris-HCl, pH 7.2 (buffer A), containing 0.5% Triton X-100 and incubated for 10 min at 20°C. The pellet was washed four times with buffer A and once with MilliQ-water and finally resuspended in ten pellet volumes (w/v) of 5 M guanidine hydrochloride (GdHCl) in buffer A followed by stirring for 20 min at 20°C to extract the S‑layer protein. The pellet obtained after centrifugation (35,000 x *g*, 30 min, 4°C) of that solution was washed once with 5 M GdHCl in buffer A and twice with buffer A. 100 mg (wet weight) of that preparation were incubated in a boiling 1% (w/v) SDS solution for 30 min with stirring. The pellet of PG(+) was washed repeatedly with MilliQ-water to remove traces of SDS. SCWP was released from the PG(+) material by treatment with 48% hydrofluoric acid for 96 h at 4°C [29].

Lyophilized PG-containing cell wall sacculi with (PG(+)) and without SCWP (PG(-)) were stored at -20°C.

### Interaction studies of SlhA variants and PG-containing cell wall sacculi

The different His_6_-SlhA variants were tested for their ability to interact with PG(+) and PG(-) following a recently published protocol [2,8]. Briefly, ~1 µg of SlhA protein (crude cell extract) was incubated at 37°C for 1 h with 0.2 mg of lyophilized PG(+) in 25 mM Tris–HCl, pH 8.0, containing 0.2% (v/v) Tween 20 (incubation buffer), in a total volume of 125 µl. The mixture was centrifuged (16,100 x *g*, 20 min, 4°C) yielding a supernatant containing unbound protein and a pellet of the insoluble cell wall components including attached protein. The pellet was washed twice, suspended in incubation buffer, and fractions were investigated by SDS-PAGE and by Western-immunoblotting using anti-His_6_ mouse antibody (Roche) (see below). Mixtures without PG were used as a control. The pellet fraction and the supernatant fraction of the reaction as well as the control (without PG) were quantified using the Li-Cor Odyssey Infrared Imaging System (Li-Cor; see below) and the value of precipitated protein in the control was subtracted from that in the reaction pellet. For comparability of datasets, the sum of the pellet fraction and the supernatant fraction of each reaction was set to 100%. Experiments were performed in duplicate and standard deviations were calculated.

### Fluorescence microscopy

The surface accessibility of displayed SlhA_EGFP and SpaA_His_6_ chimeras was analyzed directly by fluorescence microscopy and after immunofluorescence staining, respectively. *P. alvei* CCM 2051^T^ ∆*slhA* cells expressing both proteins from pSURF (see Table 1) were harvested at an optical density of OD_600_ ~0.6 and washed three times with phosphate-buffered saline (PBS). After resuspension in 100 µl of PBS, 5 µl of a penta-His Alexa Fluor 532 conjugate (Qiagen) was used and incubation was done for 3 h at 25°C on a horizontal shaker. After washing for three times, the cells were resuspended in 100 µl of PBS analyzed by fluorescence microscopy. Three μl of that suspension were mixed with 3 μl of a solution containing 0.9% molten agarose, 40 mM Tris-HCl (pH 8.0), 20 mM acetic acid, 1 mM EDTA, pH 8.0, and pre-heated to 55°C. The samples were covered immediately with glass to immobilize the cells in the solidified agarose.

The samples were monitored using a Nikon Eclipse TE2000-S fluorescence microscope at a magnification of 100 x using an oil immersion objective. In the case of the Alexa Fluor 532-labelled SpaA-His_6_, the TRITC filter block and for SlhA-EGFP, the GFP filter block were used. Pictures were taken with a Nikon Digital Sight DS-Qi1Mc camera and the NIS-Elements 3.22 imaging software.

### General and analytical methods

SDS-PAGE was carried out according to a standard protocol [30] using a Protean II electrophoresis apparatus (Bio-Rad). Protein bands were visualized with Coomassie Brilliant Blue G 250 staining reagent.

For Western-immunoblotting of proteins onto a polyvinylidene difluoride membrane (Bio-Rad) a Mini Trans-Blot Cell (Bio-Rad) was used. Detection of the His_6_-tag fused to SlhA-variants was done with the Li-Cor Odyssey Infrared Imaging System using anti-His_6_ mouse antibody (Roche) in combination with goat anti-mouse IgGIR Dye 800CW conjugate (Li-Cor). The integrated intensity of the detected bands was determined using the Li‑Cor Odyssey Application Software 3.0.21 applying automatic background subtraction. PG-containing cell wall sacculi (PG (+) and PG(-)) were analyzed for their content of aminosugars and diaminopimelic acid (DAP) using a Biochrom 30 amino acid analyzer as described elsewhere [2].

### Colony morphology, swarming and adhesion test

For imaging of the colony morphology of single cells of *P. alvei* CCM 2051^T^ wild-type, *P. alvei* Δ*slhA*, complemented mutant (*P. alvei* Δ*slhA*_comp_) and *P. alvei* Δhag, pictures were taken using a SteREO Discovery. V12 (Zeiss) microscope and an AxioCam MRc5 camera (Zeiss) after growth for 48 h at 37°C on 2% LB-plates.

To compare the adhesion of *P. alvei* CCM 2051^T^ wild-type, *P. alvei* Δ*slhA*, *P. alvei* Δ*slhA*_comp_ and *P. alvei* Δhag cells to nutrient plates, an assay based on the adhesion and agar invasion assay for yeast was used [31]. Cells were grown to OD_600_ ~1.0 and 5 µl of the culture were spotted on a 2% LB-agar plate (pH 7.0). Plates were incubated overnight at 37°C. The adhesion of the cells to the agar plates was tested by intense rinsing of the plates with distilled water using a wash bottle. Pictures were taken with a SPImager (S&P Robotics).

To test the different *P. alvei* variants for their ability to swarm on agar plates, cells were grown to the exponential growth phase (OD_600_ ~1.0) and 5 µl of each culture were spotted on 0.4% (soft agar), 1% (semi-solid agar) and 1.5% (hard agar) LB-agar plates (pH 6.0). *P. alvei* cells without the pEXALV plasmid were cultivated at 37°C for 24 h, while strains containing pEXALV were incubated at 37°C for 48° h. Pictures were taken using a SPImager (S&P Robotics).

### Congo red assay

To quantify EPS production in *P. alvei* CCM 2051^T^ wild-type and mutants, cells from 1 ml of an overnight-culture grown in LB medium were pelleted and washed once with 1 ml of LB broth. The pellet was resuspended in 1 ml of broth containing 40 µg/ml Congo red and incubated for 2 h at 37°C with shaking (250 rpm). Congo red bound to *P. alvei* cells was removed by centrifugation and unbound Congo red was determined by measuring the absorbance of the supernatant at 490 nm [32]. Data represent mean values + SD of at least four independent experiments and were analyzed by the unpaired Student’s T Test. Asterisks indicate significant differences (*, P < 0.05; **, P < 0.01; ***, P < 0.001).

To detect EPS production on plates, an overnight-culture was diluted to OD_600_ ~1.0 and 5 µl of that culture were spotted on 2% LB plates containing 40 µg/ml Congo red. Plates were incubated at 37°C for 24 h and 48 h at 25°C, red spots indicated binding of Congo red [32].

### Transmission electron microscopy (TEM)

To identify potential differences in flagellation of *P. alvei* CCM 2051^T^ wild-type, *P. alvei* Δ*slhA* and *P. alvei* Δhag, cells were grown over-night in LB medium. 30 µl of these cell suspensions were applied to Formvar- and carbon-coated 300-mesh copper grids (Agar Scientific) that were rendered hydrophilic upon glow discharge using a Pelco easiGlow apparatus (Ted Pella) for TEM. The grids were incubated for 10 min face down on the cell suspensions. Samples were fixed with 2.5% glutaraldehyde, washed three times with MilliQ-water, and stained with 1% uranyl acetate solution, pH 4.2, for 40 s [33]. Samples were investigated using a Tecnai G^2^ 20 Twin transmission electron microscope (TEM; FEI), operating at 80 keV. Pictures were taken with an FEI Eagle 4 k CCD camera (4096×4096 pixels).

### Microtitre plate assay

The ability of the *P. alvei* CCM 2051^T^ variants to form biofilm on an abiotic surface was evaluated according to Yegorenkova et al. [21]. Cultures were grown overnight in LB medium, diluted to an OD_600_ ~0.025 with sterile nutrient medium, and 1-ml aliquots were pipetted into 24-well polystyrene plates (STARLAB). The control received only the sterile nutrient medium. Cultures were incubated at 37°C for 72 h without shaking. Subsequently, planktonic cells were gently removed with a pipette and the plates were washed twice with MilliQ-water. The OD_600_ of planktonic cells was determined. Each well received 1 ml of 1% (w/v) Crystal violet and the plates were allowed to stand for 20 min at 25°C. Crystal violet solution was withdrawn with a pipette and the plates were carefully washed with MilliQ-water. Bound dye was released by adding 2.0 ml of acetone-ethanol (1:4) to the wells. Biofilm forming ability was evaluated by measuring the absorbance at 590 nm (A_590_) of the solution using a Hitachi U-2001 spectrophotometer. Crystal violet values were normalized to the corresponding absorbance of the planktonic cells (A_590_/A_600_). Data represent mean values + SD of at least four independent experiments with each four replicates and were analyzed by the unpaired Student’s T Test. Asterisks indicate significant differences (*, P < 0.05; **, P < 0.01; ***, P < 0.001).

### Confocal laser scanning microscopy (CLSM)

*P. alvei* CCM 2051^T^ variants were grown for 72 h in uncoated plastic dishes (µ-Dishes, 35 mm high; Ibidi). Prior to CLMS, the biofilm was washed with 1 ml of PBS buffer to remove planktonic cells and 1 ml of PBS buffer was added.

To visualize the cells of the biofilm, 1 ml of a solution of Hoechst 33258 (Sigma Aldrich) (10 ng/ml) was added to the biofilm and incubated in the dark at room temperature for 25 minutes. Images were taken on an inverted TCS-SP5 confocal microscope (Leica) at excitation of 356 nm and emission of 465 nm [34].

### Scanning electron microscopy (SEM)

*P. alvei* CCM 2051^T^ variants were grown as standing cultures on glass slides in cell culture dishes (uncoated, PS, 35 mm; Thermo, Fisher) containing 2 ml of LB medium. After 24 h, 48 h and 72 h respectively, the biofilm was washed with PBS to remove planktonic cells, followed by fixation with 2.5% glutaraldehyde in PBS for 4 h at 4°C. Subsequently, a dehydration series in ethanol (25%, 35%, 50%, 60%, 70%, 80%, 90%, 95%, 100% each for 7 min at room temperature) followed by drying with hexamethyldisilazane (HMDS; 33%, 66% and 100% in methanol), the glass slides were carefully removed and air-dried for several hours, were sputter-coated with 2.8-nm Au and examined at 20 keV acceleration voltage using a Inspect S50 scanning electron microscope (FEI) [35].

## Results

To investigate the role of the C-terminal SLH-domains of SlhA for cell surface attachment, different C-terminal truncations of SlhA devoid of the SP, i.e., SlhA recombinant, SlhA_12_ (lacking the SP and SLH domain three), SlhA_1_ (lacking SLH domains two and three), and SlhA w/o SLH (lacking all three SLH domains) (Figure 1A), were tested for their ability to bind to native PG-containing cell wall sacculi (PG(+)) of *P. alvei* CCM 2051^T^; which mimicked the native cell envelope.

**Figure 2:**
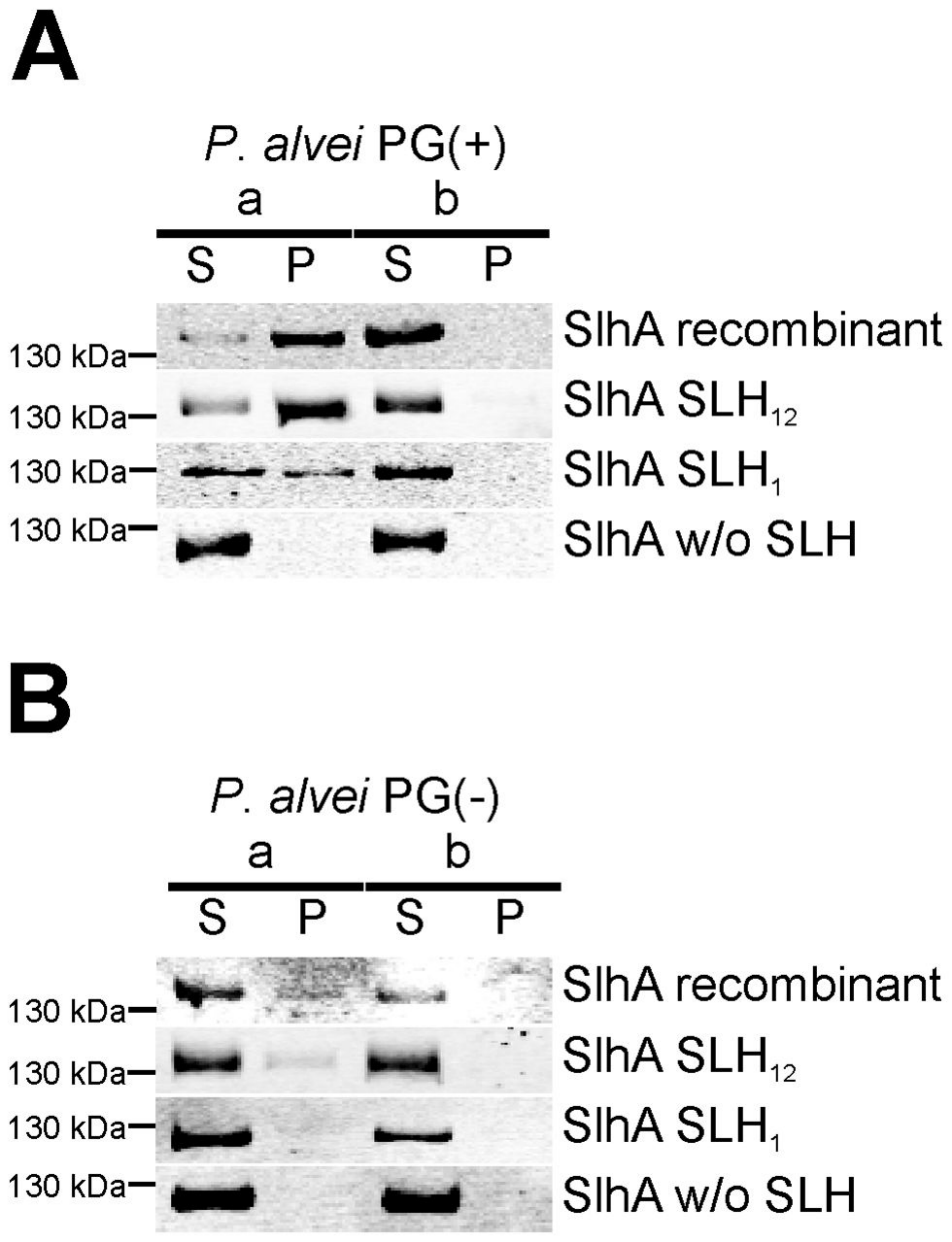
One SLH domain is sufficient for binding of SlhA to native cell wall sacculi. Binding of (A) native SlhA and SlhA truncations to PG(+) and (B) PG(-) cell wall sacculi of *P. alvei* was tested. SlhA was truncated for either one (SlhA-SLH_12_, lacking SLH domain 3), two (SlhA-SLH_1_, lacking SLH domains 2 and 3) or all three (SlhA-w/o SLH) SLH domains. Cell extracts containing the SlhA protein versions were incubated (a) with and (b) without cell wall sacculi. After incubation the reactions were centrifuged to separate cell walls (with bound protein) from unbound protein. Analysis was done by SDS-PAGE (8-10% gels) followed by Western-immunoblotting using anit-His_6_-antibody. The integrated intensity of the detected bands was determined using the Li‑Cor Odyssey Application Software 3.0.21 applying automatic background subtraction. 10 µl of each sample were loaded onto the gel. L, PageRuler^TM^ Plus Prestained Protein Ladder (Fermentas); S, supernatant; P, pellet; w/o, without. Results of the Western blots used for quantification are summarized in Table 3. The figure represents one of at least two independent repeats of the experiment.

The results from the *in vitro* binding assay as evidenced by densitometric quantification of Western-immunoblots indicated that one SLH domain of SlhA (as evidenced for the innermost SLH-domain) is sufficient for binding to native PG(+), with ~88% retention of binding observed for SlhA-SLH_12_ and decreased binding for SlhA-SLH_1_ (~44%) in comparison to the recombinant full-length protein (SlhA recombinant) (compare with Table 3). The truncated S‑layer protein devoid of all three SLH domains (SlhA-w/o SLH) was non-reactive with that material confirming the binding hypothesis (Figure 2A).

**Table 3:** Quantification of the *in vitro* binding assay of the SlhA protein and SlhA variants with truncated SLH-domains.

**Variant**	**Binding to PG(+)**	**Binding to PG(-)**
SlhA recombinant	88.2 + 12.3%	25.0 + 11.8%
SlhA-SLH_12_	77.3 + 6.9%	7.5 + 7.3%
SlhA-SLH_1_	38.8 + 13.8%	1.8 + 2.5%
SlhA-w/o SLH	0.8 + 1.1%	0.1 + 0.1%

Data are shown as mean + standard deviation from at least two independent experiments. SlhA was truncated for either SLH3 (SlhA-SLH_12_), SLH1 and SLH2 (SlhA-SLH_1_) or all three (SlhA-w/o SLH) SLH domains and was tested for binding in comparison to recombinant SlhA. Abbreviations: w/o, without.

To test, if the binding of SlhA was specific for the SCWP associated with the native PG of *P. alvei* CCM 2051^T^, the cell walls were treated with HF to release the covalently bound SCWP, yielding PG(-) cell envelope material [29]. Amino acid analysis confirmed that this material was completely SCWP-free [2]. Using that PG(-) material in the *in vitro* binding assay clearly showed that recombinant full-length SlhA and also SlhA_SLH_12_ still showed ~25.0% and ~7.5% binding capability, respectively, to PG(-), whereas SlhA_SLH_1_ showed only residual binding of ~1.8% and the truncated S‑layer protein devoid of all three SLH domains was non-reactive with PG(-) (Figure 2B, Table 3).

Summarizing, the *in vitro* experiments suggest that the innermost SLH-domain (SLH1) is sufficient for anchoring of the SlhA protein (corresponding to SlhA_32-1169_) to the native PG layer. As has already previously been reported for the S‑layer glycoprotein SpaA, the SLH-domains of SlhA have dual recognition function, both for the SCWP and for the PG [2].

### Localization of SlhA to the cell surface of *P. alvei ∆slhA*

To localize the SlhA protein on the cell surface of *P. alvei* ∆*slhA* in relation to the S‑layer protein SpaA, which is known to completely cover the bacteria as a 2D crystalline array with oblique symmetry (10), a cell surface co-display system was constructed based on a chimera made of SlhA-EGFP and SpaA-His_6_.

**Figure 3:**
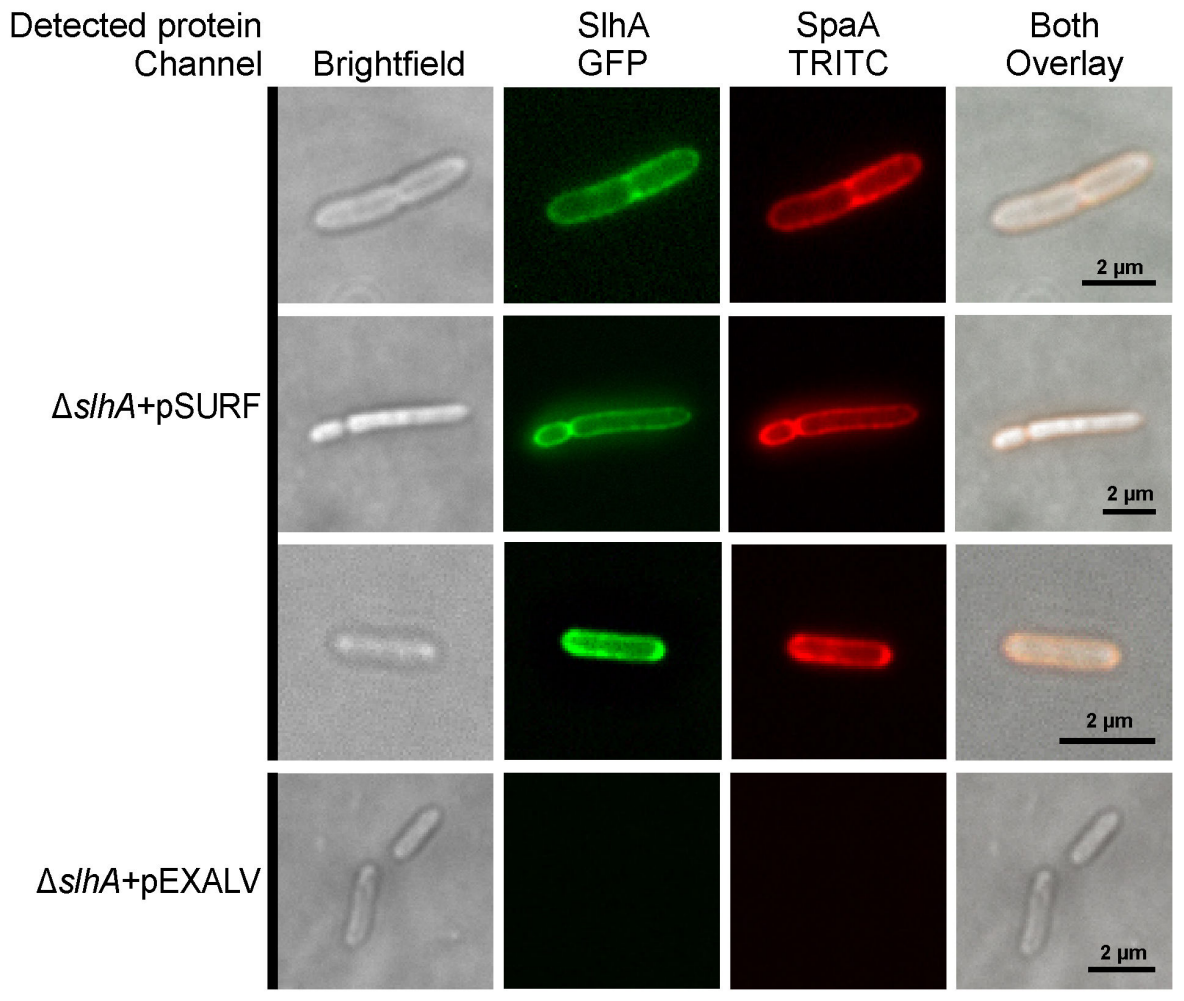
Immunofluorescence microscopy of *P. alvei* CCM 2051^T^ Δ*slh*A cells co-displaying SlhA_EGFP and SpaA_His_6_. For immunofluorescence staining of surface-located SpaA_His_6_, a penta-His Alexa Fluor 532 conjugate for direct detection of the His_6_-tagged SpaA was used. The TRITC and the GFP Long pass filter blocks were used for detection of Alexa Fluor 532 and EGFP, respectively. The upper three rows show the immunofluorescence microscopy pictures of cells harboring pSURF and co-displaying SlhA_EGFP (upper three rows, second pictures) and SpaA_His_6_ (upper three panels, third pictures). *P. alvei* CCM 2051^T^ Δ*slh*A cells harboring pEXALV are shown as a control in the fourth panel. Corresponding brightfield images of the same cells are shown on the very left and overlays are shown on the very right of each panel.

*P. alvei* ∆*slhA* cells simultaneously expressing the two tagged-proteins were analyzed by fluorescence microscopy. The appearance of a continuous fluorescent halo around the cells indicated that SlhA is located on the outermost surface of *P. alvei* ∆*slhA* cells. SpaA-His_6_ was detected by probing the cells with penta-His Alexa Fluor 532 conjugate (Figure 3, upper panel). In contrast, *P. alvei* ∆*slhA* cells expressing the empty vector did not show any fluorescence (Figure 3, lower panel).

### Knockout of *slhA* causes changes in colony morphology and bacterial adhesion to agar plates

**Figure 4:**
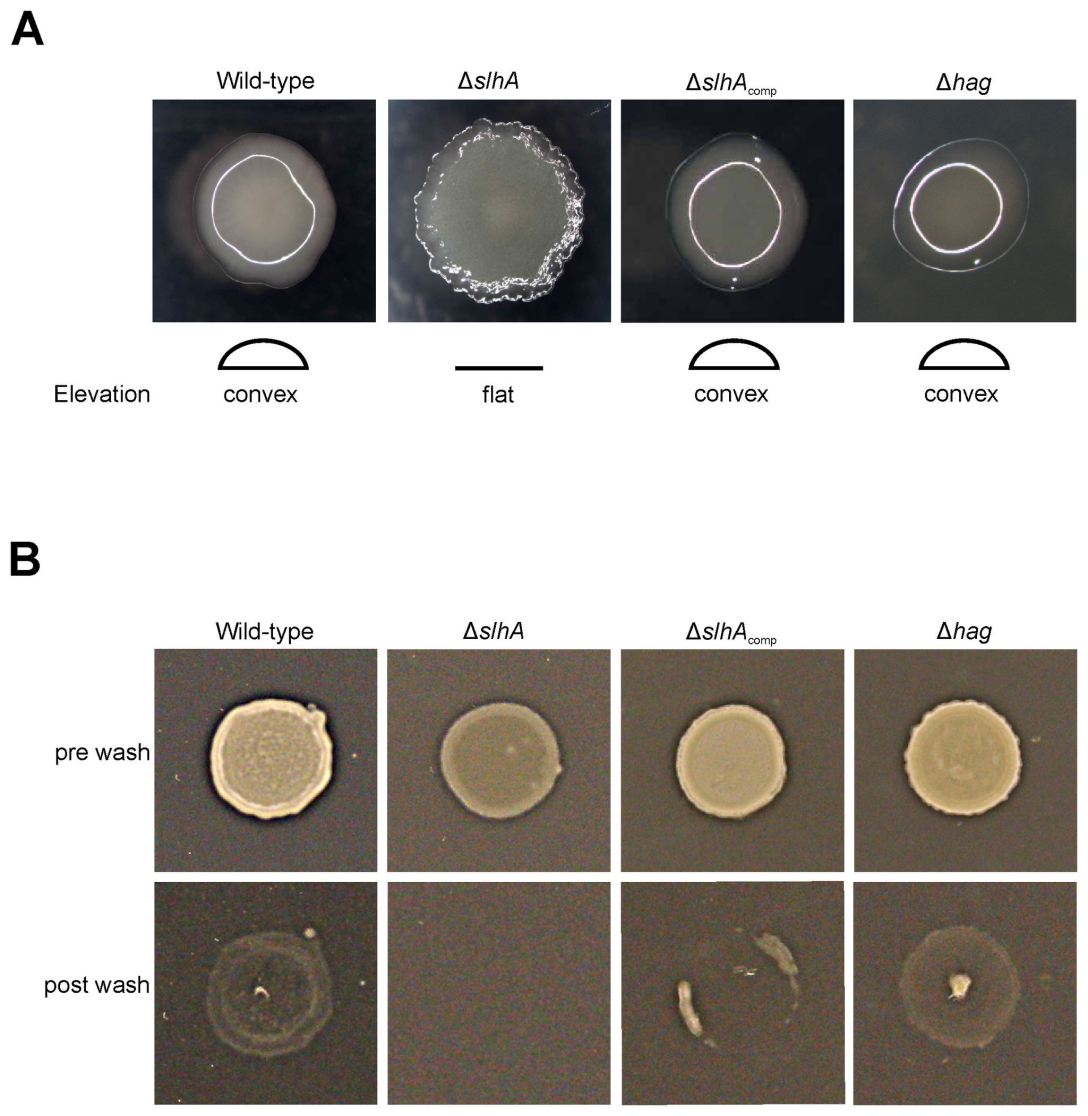
Knockout of *slhA* causes changes in colony morphology and adhesion of *P. alvei* CCM 2051^T^ cells to agar plates. (A) Colony morphology of the wild-type strain (A, first picture), *P. alvei* CCM 2051^T^ Δ*slh*A (A, second picture), *P. alvei* CCM 2051^T^ Δ*slh*A_comp_ (A, third picture) and *P. alvei* CCM 2051^T^ Δ*hag* (A, fourth picture) on LB agar plates. (B) Adhesion test of wild-type, *P. alvei* Δ*slh*A, the complemented strain and *P. alvei* Δhag. The upper panel shows the wild-type (B, upper panel first picture), *P. alvei* Δ*slh*A (B, upper panel second picture), the complemented strain ((B, upper panel third picture) and *P. alvei* Δhag (B, upper panel third picture) before washing, the lower panel shows the same strains after the washing step. The pictures represent one of four independent experiments.

To determine a putative function of the SlhA surface protein, an *slhA* knockout was created based on bacterial mobile group II intron-mediated gene disruption [26]. Reconstitution of *P. alvei* CCM 2051^T^ *slhA*::Ll.LtrB (*P. alvei* Δ*slh*A) was done by plasmid-based SlhA expression. The Δ*slh*A mutant clearly differed from the wild-type bacterium in colony morphology when grown on 2% LB-agar plates (Figure 4A). The colonies of the SlhA knockout were non-adhesive, flat and frayed, while those of the wild-type, the complemented bacterium and a flagella knockout (*P. alvei* Δhag) were sticky, convex and circular (Figure 4A). *P. alvei* Δhag was included in this study for comparison based on our assumption that SlhA might be involved in bacterial motility.

An adhesion test, in which *P. alvei* CCM 2051^T^ wild-type, *P. alvei* Δ*slh*A, *P. alvei* Δ*slh*A_comp_ and *P. alvei* Δhag cultures were spotted onto agar plates, incubated over night at 37°C and washed thoroughly, clearly showed an increased adhesion capability of wild-type, *P. alvei* Δ*slh*A_comp_ and *P. alvei* Δhag cells compared to *P. alvei* Δ*slh*A cells (Figure 4B). Pictures taken immediately before the washing step showed identical spot sizes for all variants, with *P. alvei* Δ*slh*A being more transparent, possibly due to different colony morphology (Figure 4B, upper panel). After the washing step, the *P. alvei* wild-type, *P. alvei* Δ*slh*A_comp_ and *P. alvei* Δhag spots remained visible, while the *P. alvei* Δ*slh*A colony has been completely removed (Figure 4B, lower panel). This clearly indicated differences in the adhesion capability to 2% LB-agar plates of *P. alvei* CCM 2051^T^ wild-type, *P. alvei* Δ*slh*A_comp_ and *P. alvei* Δhag in comparison to *P. alvei* Δ*slh*A.

### Knock-out of *slhA* does not affect flagellation of *P. alvei*

**Figure 5:**
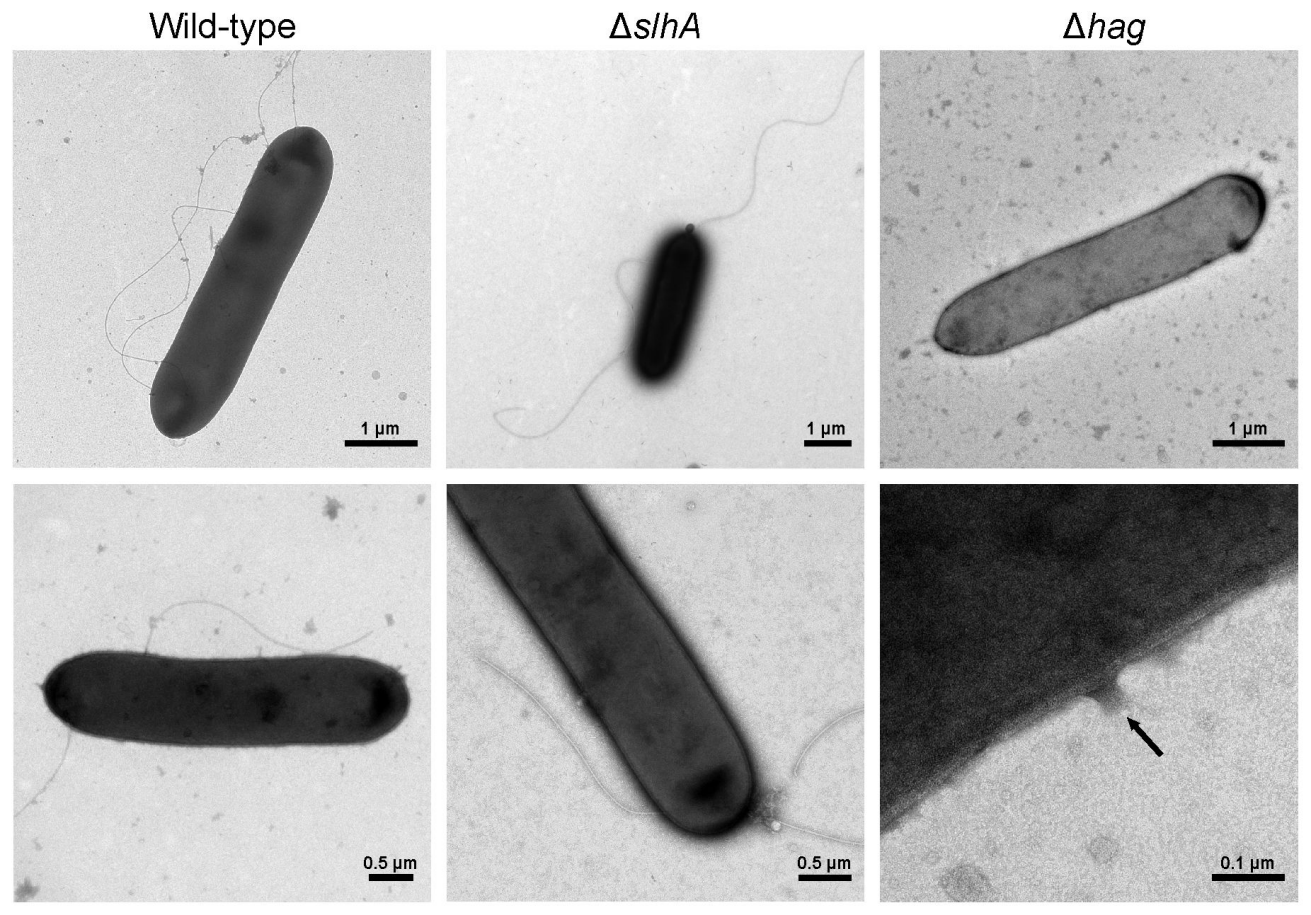
Knockout of *slhA* does not alter flagella production of *P. alvei* CCM 2051^T^ cells. Electron microscopic view of *P. alvei* CCM 2051^T^ wild-type (first column), Δ*slh*A (second column) and Δ*hag* cells (third column). Flagella are clearly visible for wild-type and Δ*slh*A cells but no flagella are present for Δ*hag* cells.

Following up the effect of SlhA on bacterial motility, it was investigated by TEM, if *P. alvei* CCM 2051^T^ wild-type, *P. alvei* Δ*slhA* and *P. alvei* Δhag cells grown in LB broth showed differences in flagellation. To avoid shear forces, cell suspensions of the overnight cultures were directly used for TEM analysis. According to the TEM evidence on both *P. alvei* CCM 2051^T^ wild-type and *P. alvei* Δ*slhA* cells flagella are clearly visible, but are absent in *P. alvei* Δhag cells. Thus, there is no evidence that SlhA would be directly involved in the production or formation of flagella (Figure 5).

### *P. alvei* CCM 2051^T^ Δ*slh*A cells lose the ability to swarm on LB-agar plates

**Figure 6:**
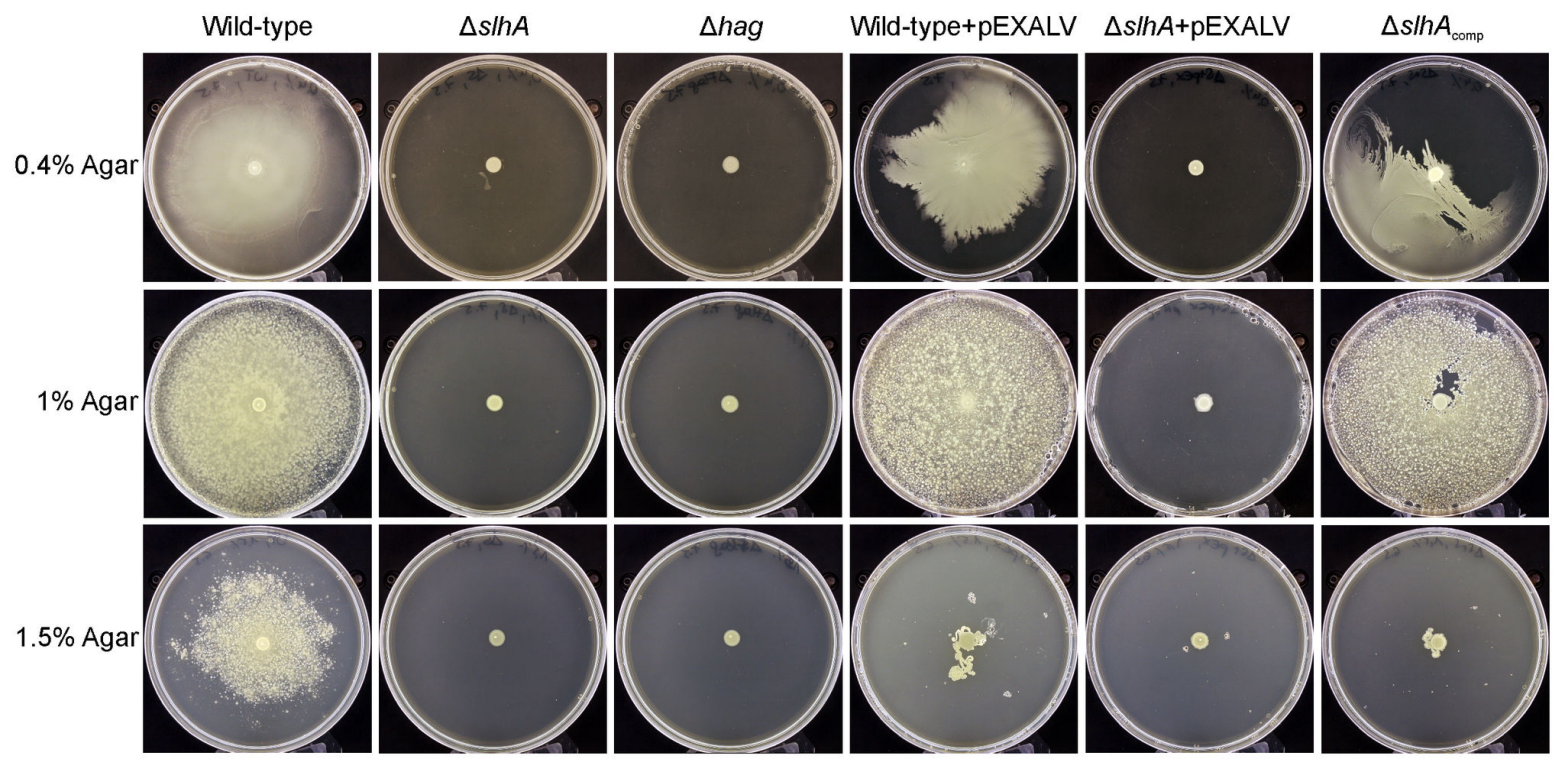
*P. alvei* CCM 2051^T^ Δ*slh*A cells lose the ability to swarm on LB-agar plates. The upper panel shows swarming cells of wild-type (first column), *P. alvei* Δ*slh*A (second column), *P. alvei* Δhag (third column), wild-type (pEXALV) (fourth column), *P. alvei* Δ*slh*A (pEXALV) (fifth column) and the complemented strain *P. alvei* Δ*slh*A_comp_ (sixth column) on 0.4% (upper panel), 1% (middle panel) and 1.5% (lower panel) LB-agar plates. The pictures represent one of three independent experiments.

Since *P. alvei* cells are known to vigorously swarm on agar plates [13], we were interested to know, if swarming was changed in the Δ*slh*A mutant. Swarming on 0.4%, 1% and 1.5% LB-agar plates was completely abolished for *P. alvei* Δ*slhA*, while the complemented strain *P. alvei* Δ*slhA*_comp_ was able to swarm as effectively as the wild-type bacterium (Figure 6). Considering our observation of the complemented bacterium requiring 48 h instead of 24 h incubation time to swarm as efficiently as the *P. alvei* wild-type, it was tested, if the pEXALV expression vector had an influence on bacterial swarming. In fact, *P. alvei* carrying empty pEXALV vector needed longer (48 h) to swarm to the level of the vector-free wild-type bacterium.

Thus, to investigate, if *P. alvei* cells carrying pEXALV have a slower growth phenotype (extended lag-phase) compared to the wild-type, growth curves were recorded for the *P. alvei* variants. Comparison of the generation times showed almost identical values for all tested *P. alvei* variants (data not shown).

Since the expression vector pEXALV affects the lag-phase and, consequently, the swarming ability of *P. alvei* CCM 2051^T^ on agar plates (48 h versus 24 h), but not the generation time during exponential growth, loss of swarming motility can be interpreted as a direct consequence of knocking out *slhA*. Since also, as demonstrated for *P. alvei* Δ*slhA*, *P. alvei* Δhag was not able to swarm on 0.4%, 1% or 1.5% LB agar plates we assume that the flagella might be non- functional in the *slhA* knockout background under the used conditions (Figure 6).

### Knockout of *slhA* decreases biofilm formation of *P. alvei* CCM 2051^T^ cells

**Figure 7:**
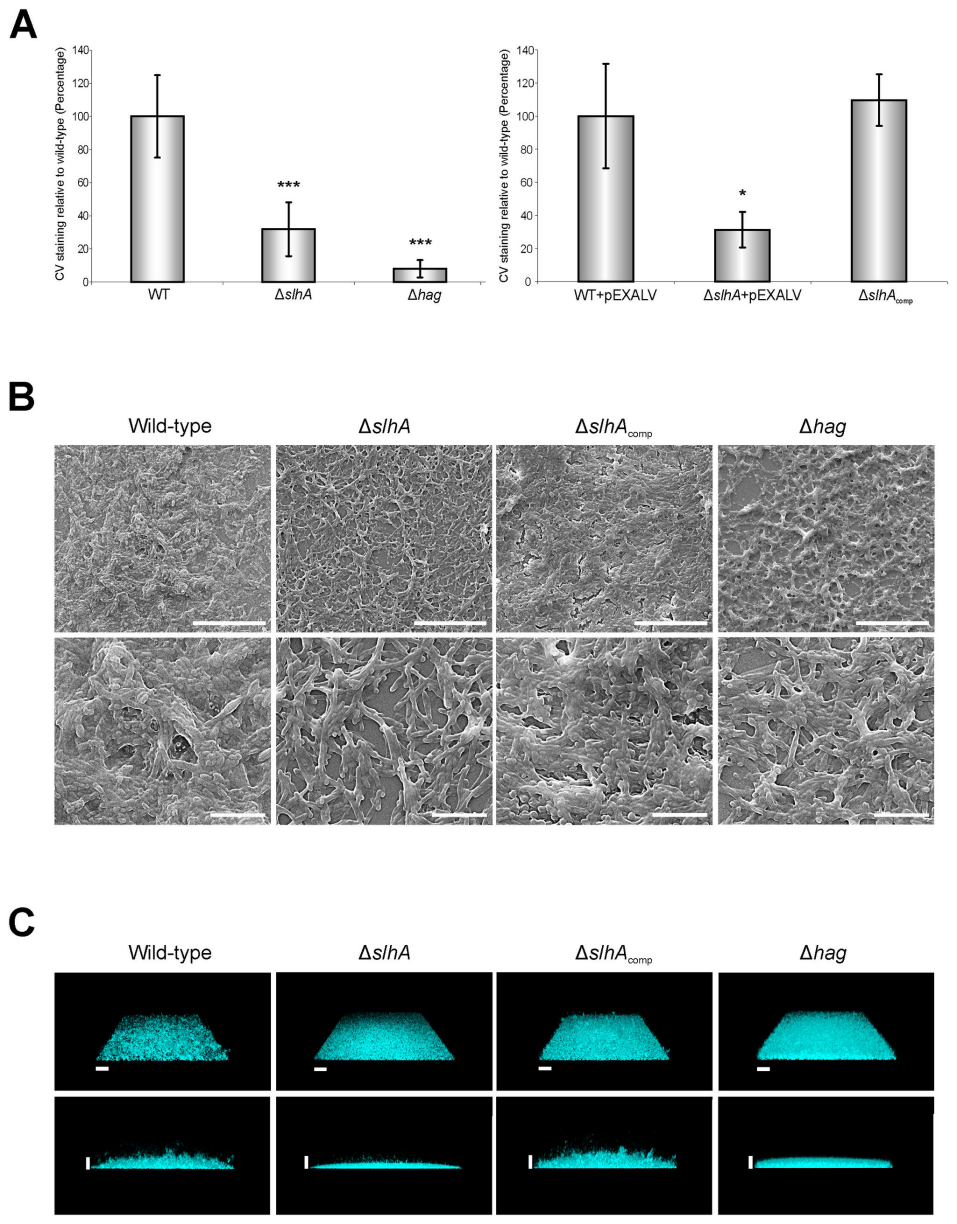
Knockout of *slhA* and *hag* decreases biofilm formation of *P. alvei* CCM 2051^T^ cells. (A) Evaluation of the ability of cells of *P. alvei* CCM 2051^T^ wild-type, Δ*slh*A, Δ*hag*, wild-type (pEXALV), Δ*slh*A (pEXALV) carrying the pEXALV vector, and the complemented strain *P. alvei* Δ*slh*A_comp_ for biofilm formation using Crystal violet (CV) staining. Data represent mean values + SD of at least four independent experiments with each four replicates and were analyzed by the unpaired Student’s T Test. Asterisks indicate significant differences (*, P < 0.05; **, P < 0.01; ***, P < 0.001). (B) SEM analysis of *P. alvei* CCM 2051^T^ wild-type, Δ*slh*A, Δ*hag* and the complemented strain Δ*slh*A_comp_ showing an overview and enlarged view of the biofilm. Size bars are 20 µm for the upper panel and 5 µm for the lower panel. (C) CSLM analysis of *P. alvei* CCM 2051^T^ wild-type, Δ*slh*A, Δ*hag* and the complemented strain Δ*slh*A_comp_ stained with Hoechst 33258 showing a diagonally above view (upper panel) and a side view (lower panel) of a three day biofilm. Size bars are 20 µm.

In a next step, it was investigated, if SlhA would also affect biofilm formation of *P. alvei* CCM 2051^T^. Biofilm formation of *P. alvei* wild-type, *P. alvei* Δ*slhA* and *P. alvei* Δhag was monitored at 37°C for 72 h using a crystal violet-based microtiter assay [21]. Since pEXALV was shown to influence bacterial growth (see above), *P. alvei* wild-type and *P. alvei* Δ*slhA* cells, vector-free and carrying pEXALV, respectively, were investigated in two independent experiments. Crystal violet values were normalized to the corresponding absorbance of the planktonic cells (A_590_/A_600_). In both experimental settings, wild-type cells were set to 100% (wild-type: 100% +24.9%, *P. alvei* wild-type (pEXALV): 100% +8.9%). *P. alvei* Δ*slh*A cells showed 31.9% +16.3%, *P. alvei* Δhag cells 8.0% +5.2% of biofilm formation compared to the wild-type. *P. alvei* Δ*slh*A (pEXALV) retained 31.3 +10.8%, while complemented *P. alvei* Δ*slh*A_comp_ showed 109.7% +15.6% of biofilm formation (Figure 7A).

Thus, knocking out of *slhA* caused a significant decrease in biofilm formation, being 3.1-fold less for cells without pEXALV and 3.2-fold less for cells carrying pEXALV in comparison to the wild-type bacterium. Plasmid-based expression of SlhA complemented biofilm formation to wild-type levels. Knocking out of *hag* resulted in 12.5-fold less biofilm formation compared to the wild-type level according to the microtiter assay.

SEM and CLSM confirmed the results of the microtiter assay. For each technique, three biological replicates were analyzed. The SEM pictures taken after 72 h of biofilm development on glass slides revealed a biofilm with a higher cell density for the wild-type and *P. alvei* Δ*slh*A_comp_ compared to *P. alvei* Δ*slh*A and *P. alvei* Δhag (Figure 7B). The *P. alvei* Δ*slh*A biofilm showed a reduced content of slimy matrix components, probably due to reduced EPS formation.

The 3D structure of 72 h wild-type, *P. alvei* Δ*slh*A, *P. alvei* Δ*slh*A_comp_ and *P. alvei* Δhag biofilms was visualized by CLSM after staining with Hoechst (Figure 7C). Biofilms of *P. alvei* Δ*slh*A and *P. alvei* Δhag were comparable in height, structure and density. Either strain showed preferentially a carpet-like appearance, with few cell-layers of ~5 µm (Δ*slh*A) and ~10 µm (Δ*hag*) respectively. Tower-like structures were produced by the wild-type (~3 µm, height) and the *P. alvei* Δ*slh*A_comp_ (~34 µm, height) biofilms. This supported the results of the microtiter assay.

Summarizing, the biofilm of the wild-type is ~6-fold thicker than that of Δ*slh*A and ~3-fold thicker than that of Δ*hag*, according to CLSM analysis. CLSM analysis of Δ*slh*A_comp_ revealed again a slightly increased biofilm formation compared to the microtiter assay. In conclusion, the wild-type and the complemented strain showed by far the best ability for biofilm formation according to three different techniques (microtiter assay, CLSM; SEM). The Δ*slh*A and Δ*hag* mutant strains both showed significantly reduced biofilm formation.

### Knock-out of *slhA* decreases Congo red-staining of *P. alvei* CCM 2051^T^ cells

Given that colonies of the *P. alvei* CCM 2051^T^ wild-type were found to be sticky compared to *P. alvei* Δ*slhA*, the composition of the extracellular matrix could have been altered in the *slhA* mutant.

**Figure 8:**
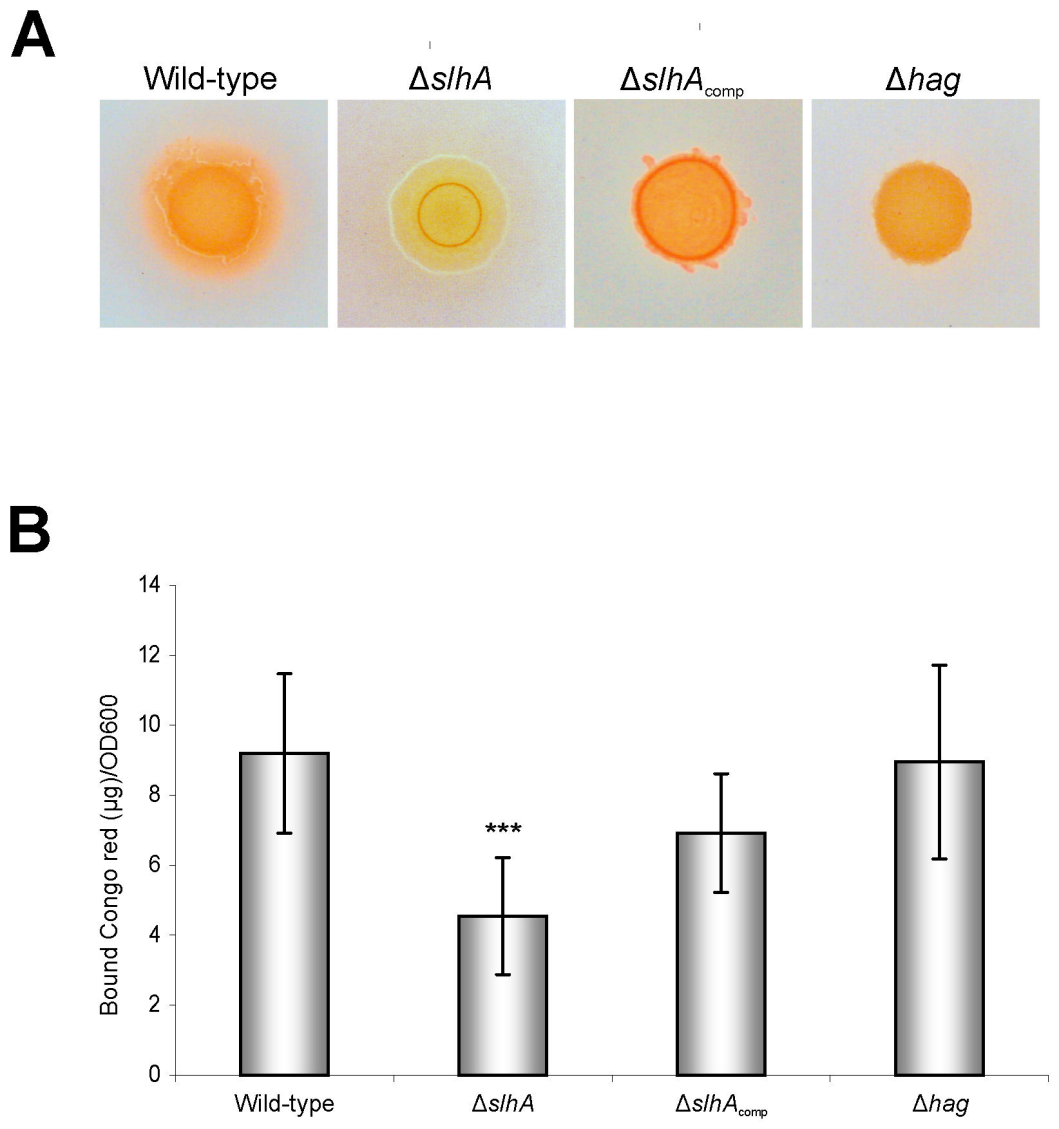
Knockout of *slhA* decreases Congo red staining of *P. alvei* CCM 2051^T^ cells. (A) Congo red plates of *P. alvei* CCM 2051^T^ wild-type, Δ*slhA*, Δ*slh*A_comp_ and Δ*hag* cultures. (B) Quantification of Congo red-binding in planktonic cultures at 37°C. Data represent mean values + SD of at least four independent experiments and were analyzed by the unpaired Student’s T Test. Asterisks indicate significant differences (*, P < 0.05; **, P < 0.01; ***, P < 0.001).

*P. alvei* CCM 2051^T^ wild-type, *P. alvei* Δ*slhA*, *P. alvei* Δ*slhA*_comp_ and *P. alvei* Δhag were tested for their EPS production by spotting the cells on Congo red (CR)/LB agar plates. CR accumulates on the colony surface over time only when EPS is produced [36]. Upon incubation of the plates for 24 h at 37°C followed by incubation for 48 h at 25°C, colonies of the wild-type, Δ*slh*A_comp_, and Δ*hag* strain had a reddish appearance, indicative of the presence of CR-reactive EPS on either variant. In comparison, spotted Δ*slhA* cells showed decreased adsorption of CR (Figure 8A). Quantification of EPS production of planktonic cells showed that the wild-type bound 9.2 +2.3 µg CR/OD_600_, while Δ*slhA* bound 4.5 +1.7 µg CR/OD_600_ and Δ*slhA*_comp_ bound 6.9 +1.7 µg CR/OD_600_ (Figure 6B). CR binding ability of Δ*hag* of 9.0 +2.8 µg compared well to that of the wild-type (Figure 8B). The value of 49% of CR binding of Δ*slhA*, in relation to 97% CR binding of Δ*hag* compared to the wild-type bacterium, might be indicative of altered EPS production in the Δ*slhA* mutant.

## Discussion

Gram-positive bacteria synthesize a thick cell wall, which serves as an assembly scaffold for the surface display of polypeptides, capsular polymers, and wall teichoic acids [37]. These cell surface molecules strongly influence the physicochemical and functional properties of bacterial cells and represent an important interaction zone with the environment. For cell surface display, many proteins are endowed with terminal SLH-domains that mediate non-covalent surface attachment via interaction with pyruvylated moieties of a SCWP [4,8,9,38-43]. For this interaction, especially the TRAE motif that is located at the beginning of the α-helix within these domains has been reported to be critical [1,2,8]. Functional variations of that four amino acid motif have been shown for *T* *. thermosulfurigenes* EM1 [8] and *P. alvei* CCM 2051^T^, where the motifs TVEE and TRAQ are involved in anchoring of the S‑layer protein SpaA [2], and the motifs SRGE, VRQD, and LRGD are likely to be critical for anchoring of SlhA (this study). In contrast to the requirement of three functional SLH-domains as SCWP targeting modules in the cell envelope of *T* *. thermosulfurigenes* EM1 [8] or *Bacillus anthracis* [9], and four in *Lysinibacillus* *sphaericus* CCM 2177 [44], *P. alvei* CCM 2051^T^ has obviously elaborated a variation of that surface display mechanism. We have shown recently that for surface display of the S‑layer protein of that bacterium, two functional SLH domains are sufficient [2]; for the SlhA protein characterized in the course of this study, even one SLH-domain (as tested for the innermost SLH-domain, SLH1) can mediate attachment *in vitro*, with a binding capacity to PG(+) of still 38.8 ±13.8%. This reduced requirement of SLH-domain/SCWP interactions in *P. alvei* CCM 2051^T^ might be compensated by the simultaneously occurring PG/SCWP interaction which could be clearly demonstrated for the two investigated *P. alvei* CCM 2051^T^ proteins SpaA [2] and SlhA (this study). Comparison of the data from *in vitro* binding assays for the said *P. alvei* proteins indicated that binding to PG(-) was still detected for SlhA lacking the third SLH domain (SLH_12_; 7.5 ±7.3%), and even for the SlhA variant additionally lacking the second SLH-domain weak binding was possible (SLH_1_; 1.8 ±2.5%). In contrast, for the S‑layer protein SpaA no binding to PG(-) could be detected for neither the single, nor the double or the triple SLH mutant [2]. Thus, it seemed as if SLH-domain 3 of SlhA played a minor role in the binding to PG. This might, on the other hand, enable the tri-amino acid motif RGD present in that SLH-domain to be involved in host-cell integrin binding [45].

Considering the full coverage of the *P. alvei* CCM 2051^T^ cell surface with a closed 2D crystalline SpaA S‑layer lattice with oblique symmetry [26], it was important to verify the predicted cell surface localization of SlhA *in vivo*. This was approached by constructing a cell surface co-display system comprised of an SlhA-EGFP chimera and the His_6_-tagged S‑layer protein SpaA. This strategy was based on the previous demonstration that His_6_-tagged S‑layer protein gets integrated into the native SpaA-glycoprotein lattice *in vivo* and, thus, surface exposed [10]. The fluorescence signals obtained directly from SlhA_EGFP as well as from SpaA_His_6_ after immunofluorescence staining clearly demonstrated surface co-display of the proteins on *P. alvei* CCM 2051^T^ cells (compare with Figure 3). For this experiment, *P. alvei* CCM 2051^T^ Δ*slh*A cells were used because of improved transformation efficiency in comparison to the wild-type cells. This is a reliable approach, since it is known that deletion of *slhA* does not affect localization of SpaA (B. Janesch, unpublished data).

While it has been assumed previously that in S‑layer-covered bacteria the S‑layer would be the exclusive (glyco) proteinaceous layer on the cell surface, these data corroborate recent findings with *Bacillus anthracis* [46] and *Bacillus cereus* G9241 [47], where in addition to the S‑layer, S‑layer-associated proteins were found (BSLs; *Bacillus* S-layer associated proteins). While both S‑layer proteins of *B. anthracis* and BSLs bind the same SCWP, their deposition on the cell surface was found to be clearly confined. For example, the murein hydrolase BslO is targeted to septal PG zones, where it catalyzes the separation of daughter cells, while Sap S‑layer patches were not observed at the septa [46]. Regarding the co-display of SlhA and SpaA on *P. alvei* CCM 2051^T^ cells, the distribution of SlhA over the cell surface has not been investigated in detail. However, due to the uniform fluorescence signal of the SlhA_EGFP chimera, it is conceivable that it would be regularly integrated in or associated with the S‑layer lattice (Figure 3).

To unravel a putative function of the novel SlhA surface protein of *P. alvei* CCM 2051^T^, a Δ*slhA* mutant was created and its phenotype as well as distinct cell surface mediated phenomena, such as swarming motility and biofilm formation, were compared to the wild-type bacterium. According to our data SlhA is not essential for the bacterium but affects its life-style. This mutant allowed, for the first time, investigating the functional relevance of a predicted cell surface protein in that bacterium. In contrast, it has been impossible to inactivate *spaA* (B. Janesch, unpublished data) implying that the S‑layer is essential for cell envelope integrity of *P. alvei* CCM 2051^T^. Since flagella are considered a prerequisite for bacterial swarming, a flagella mutant (Δ*hag*) was created for phenotype comparison [18,48]. The first obvious difference between the wild-type and the Δ*slhA* mutant was a significant change in colony morphology from a sticky, convex and circular type into a non-adhesive, flat and frayed type (compare with Figure 4A), while colonies of Δ*hag* showed wild-type morphology. Loss of stickiness of Δ*slhA* could be confirmed in an agar adhesion assay (Figure 4B). Flagellation was visualized by TEM using negatively-stained, intact wild-type, Δ*slh*A and Δ*hag* cells that were treated very carefully to avoid damage of flagella. Peritrichous flagella could be detected for wild-type and Δ*slh*A cells (Figure 5). As expected, for Δ*hag*, only cells without flagellation were found, however on some cells, remnants of a hook were visible (Figure 5, arrow). In addition, the swarming motility on 0.4%, 1% and 1.5% LB agar plates as typical of *P. alvei* cells [11,12] was abolished in either mutant (Figure 6). A lack of surfactant for reducing the surface tension between the substrate and the bacterial cell, thereby permitting spreading over surfaces, comparable to e.g., rhamnolipid of *Pseudomonas* or surfactin of *Bacillus* species, might be an explanation for abolished swarming but not for a lack in swimming ability [16,49]. Based on the findings of Cohen et al. [13], swimming should be possible at agar concentrations below 0.5%. Since we could not detect any movement for Δ*slhA* and Δ*hag*, neither swimming nor swarming, at the tested agar concentration, it is conceivable that non-functional flagella and not a missing surfactant cause the lack in motility. It is important to note that, while no surfactant has been reported for *P. alvei* CCM 2051^T^ so far, surfactants have been found in other *P. alvei* strains. *P. alvei* LMD 50.12 is known to produce a layer of lubricating fluid for movement on hard surfaces [13] and in *P. alvei* ARN63, a biosurfactant, characterized as lipopeptide derivative, lowers the surface tension of media [50]. The presence of an ortholog of the *sfp* gene of *Bacillus subtilis* required for production of the lipoheptapeptide surfactin, on the genome of *P. alvei* CCM 2051^T^ (identity: 42%) [51], might be a further indication of a surfactant in this bacterium.

Knocking-out of *slhA* and *hag* translated into *P. alvei* CCM 2051^T^ cells with an impaired biofilm life-style according to Crystal violet assay [15,17,18]. While the Δ*slh*A mutant was 3.1-fold less efficient in biofilm formation compared to the wild-type, biofilm formation could be fully complemented by plasmid-encoded SlhA expression (Figure 7), indicating that SlhA directly correlated with biofilm formation without potential polar effects of the mutant additionally being involved. The Δ*hag* mutant showed 12.5-fold less efficiency to form biofilm compared to the wild-type indicating the importance of functional flagella for biofilm formation in *P. alvei* CCM 2051^T^. Crystal violet data were strongly supported by SEM and CLSM analyses. SEM images of wild-type and Δ*slhA*_comp_ showed a thick biofilm with cells embedded in a slimy matrix. In contrast, *P. alvei* Δ*slhA* and Δ*hag* showed a flat and thin biofilm, with the cell-covering material missing in Δ*slhA*, but not in Δ*hag*. CLSM confirmed reduced biofilm formation for Δ*slhA* and Δ*hag* compared to the wild-type, with wild-type and Δ*slhA*_comp_ forming tower-like structures, while the Δ*slhA* and Δ*hag* formed thin carpet-like biofilms.

Since the EPS matrix is generally an important factor in biofilm formation, *P. alvei* CCM 2051^T^ wild-type, Δ*slh*A, Δ*hag*, and Δ*slhA*_comp_ cells were analyzed for their EPS production upon spotting on LB plates containing CR in comparison to the respective planktonic cells (Figure 8A). The adsorption of CR has previously been shown to have a positive correlation with the presence of EPS in *Pseudomonas aeruginosa* [52,53], *Salmonella enterica* serovar Typhimurium, and *E. coli* [54]. The Δ*slh*A mutant showed only half (49%) of EPS production compared to the wild-type (Figure 8B). This might also affect the loss of stickiness of the Δ*slh*A colonies. Lauriano et al. showed that exopolysaccharide expression in *Vibrio cholerae* is regulated along a flagellum-dependent pathway [55]. They identified a sodium-driven flagellar motor as an essential component of the signaling pathway, because mutations in the motor abolished EPS production, biofilm formation and *vps* gene transcription. Cairns et al. recently showed that the level of DegU~P gets increased via the sensor kinase DegS due to an inhibition of flagellar rotation, which is an essential regulatory mechanism required for biofilm formation in *Bacillus subtilis* thereby supporting the importance of the flagellum for signal transduction purposes [56]. We hypothesize that SlhA might be involved in signal transduction based on its demonstrated effect on the motility, EPS production, and biofilm formation of *P. alvei* CCM 2051^T^ cells (this study) in conjunction with a missing link in the requirement of functional flagella for signal transduction and biofilm formation as proposed by others (see above). While there have so far been no reports on SLH domain-containing proteins (such as SlhA) functioning as receptors or their involvement in signaling cascades within the bacterial cell that could support this hypothesis, the question of how a missing or decreased flagellar rotation is sensed by the cell is still open. Considering that SlhA contains, in addition to the C-terminal SLH-domains, a galactose-binding module (CBM6; compare with Figure 1) typical of proteins binding to specific ligands, the option of SlhA functioning as a receptor remains possible. Since *P. alvei* Δhag showed abolished biofilm formation, despite production of EPS at wild-type level, we concluded that functional flagella are important for *P. alvei* CCM 2051^T^ biofilm formation. One might speculate that SlhA is involved in the signal transduction that leads to the activation of flagellation. A defect in flagellation might then lead to decreased EPS expression and reduced biofilm formation. Thus, in the future, it will be interesting to investigate the detailed composition of the EPS matrix and, especially, if SlhA is part of the biofilm matrix. There are several examples of EPS matrices known that harbor adhesive proteins. For instance, the *Staphylococcus aureus* matrix harbors biofilm-associated proteins that are anchored to the cell wall and serve to hold cells together, *Bacillus subtilis* synthesizes the EPS protein TasA and *E. coli* synthesizes the *curli* protein, with both proteins being critical for biofilm formation. Further, in *Pseudomonas aeruginosa* several surface proteins contribute to biofilm formation. Additionally lectin-binding proteins that facilitate cell-matrix or cell-cell interactions within the biofilms have been found [19].

Besides the effects of *P. alvei* CCM 2051^T^ SlhA on colony morphology, bacterial swarming, and biofilm life-style, several other functions remain open. One obvious function would be that of a surface-displayed enzyme; examples are known for EPS biofilm matrices [57]. Thus, based on some homologies of SlhA to putative amylo-pullulanases, α-amylases or pullulanases according to a BLAST search, we tested recombinant SlhA for the ability to hydrolyze starch and pullulan [58], however, without success. To extend the substrate range, *P. alvei* wild-type and *P. alvei* Δ*slhA* were tested for differences in their carbohydrate metabolism using an API^®^ 50 CH test (bioMérieux), but no differences were detected either. Thus, the question, if SlhA exerts also an enzymatic activity still remains open.

Summarizing, we showed in this study that SlhA is a novel, SLH-domain containing cell surface protein of *P. alvei* CCM 2051^T^ that is co-displayed with the S‑layer lattice. For cell-envelope anchoring of SlhA *in vitro*, the innermost SLH-domain is sufficient. It is conceivable that the motifs SRGE, VRQD, and LRGD located in the SLH-domains 1 to 3 are critical for anchoring. The SLH-domains recognize both a pyruvylated SCWP and the PG. Knocking-out of SlhA causes drastic phenotypic changes concerning colony morphology, swarming motility on agar plates, and biofilm formation. In contrast, a mutation that disrupted flagella synthesis did neither show altered EPS production nor colony morphology but impaired biofilm formation. We conclude that SlhA possibly plays an important, still unknown role in signal transduction that leads to EPS expression. Further, a complex relationship between motility, EPS production, and biofilm formation seems to exist.
